# Misophonia Levels and Care Behaviours in Intensive Care Nurses: A Descriptive, Cross‐Sectional and Relational Study

**DOI:** 10.1002/nop2.70504

**Published:** 2026-04-03

**Authors:** Zuhal Gülsoy, Tuba Karabey

**Affiliations:** ^1^ Sivas Cumhuriyet University Suşehri Health College Nursing Department Sivas Turkey; ^2^ Gaziosmanpasa University Faculty of Health Science Tokat Turkey

**Keywords:** care behaviours, intensive care, misophonia, nurses

## Abstract

**Background and Objectives:**

Misophonia is a condition that causes abnormal emotional reactions to certain environmental sounds. The aim of this study was to investigate whether there is a relationship between the responses of nurses working in intensive care units to these sounds and the care provided to the patient.

**Methods:**

Personal Information Form, Misophonia Scale and Caring Behaviours Scale‐24 were used to collect data. Mann Whitney *U* test and Kruskall Wallis test were used to analyse the data, and pearson correlation coefficient was used to examine the relationship between variables. The significance level was taken as *p* < 0.05.

**Results:**

It was determined that the mean scores of the participants on the Caring Behaviours Scale‐24 and the Misophonia Scale were moderate. A moderate negative correlation was found between the Caring Behaviours Scale‐24 and the Misophonia Scale.

**Conclusions:**

In the light of the data we obtained from the study, it is possible to say that nurses exhibiting misophonic behaviours may be adversely affected by the sounds in the intensive care environment and this situation may negatively affect their care behaviours.

**Relevance to Clinical Practice:**

Nurses with misophonic symptoms should be identified and their personal coping strategies should be strengthened. In this way, the quality of patient care and patient safety can be improved.

**Patient or Public Contribution:**

Descriptive and cross‐sectional study. After obtaining the necessary permissions for the study, the researcher met with the intensive care nurses in the nurses' work environment, provided information about the study, and obtained written consent forms from those who volunteered to participate in the study. The relevant forms were delivered to the intensive care nurses in envelopes to be filled in and were returned in a sealed envelope at the end of the 2‐day filling period. The study adhered to the STROBE checklist.

## 1 Introduction

Misophonia is a disproportionate emotional response to everyday sounds created by other people, animal sounds, and repetitive sounds such as keyboard and pen tapping (Bernstein et al. 2013; Edelstein et al. 2013; Schröder et al. 2013; Kumar et al. 2014; Erfanian et al. 2017, 2019; Schneider and Arch 2017). Actions such as breathing, wheezing, chewing, eating, slurping, lip smacking, pen tapping, touching, or cracking knuckles have been defined as some situations that cause a misophonic reaction (Schröder et al. 2013; Kumar et al. 2014; McKay et al. 2018; Sanchez and Silva 2018). Again, in many studies, misophonia has been defined as the emotional reactions experienced by people. These are distress, discomfort, anxiety, anger, disgust, hatred, and sometimes severe reactions that can lead to loss of control (Schröder et al. 2013; Kumar et al. 2014; Boyce 2015; Dozier 2015; Wu et al. 2014). Misophonia is a condition that can negatively affect the quality of life and can negatively affect work and social life (Yılmaz and Hocaoğlu 2021). In their study investigating the frequency of misophonia symptoms, Wu et al. (2014) reported that 22.8% of the participants were sensitive to repetitive environmental sounds such as the sound of tapping a pencil on the table, and 22.8% to chewing, swallowing, smacking sounds, and stamping their feet. In the same study, it was found that 52.1% of the participants with clinically significant misophonia symptoms had their functionality significantly negatively affected in the work and school environment.

Intensive care units (ICUs) are environments that provide services to critically ill patients and where people are exposed to many different types and intensities of sound in the work environment. In parallel with the variability of bed occupancy rates in ICUs, the increase in the number of personnel and medical equipment used causes the noise level and variety in the unit to increase (Lewis and Oster 2019; Cvach et al. 2020). When intensive care workers are exposed to loud and different alarms for a long time, symptoms such as fatigue, sensory overload, boredom, unwillingness to hear, and desensitisation, that is, not responding to alarms, occur (Dursun Ergezen and Kol 2019; Suba et al. 2019; West et al. 2014). In this context, instead of creating a safer environment for patients, alarms, contrary to expectations, can cause the work environment to be more stressful, cause sensory overload in nurses, disrupt thought processes, and jeopardize the quality of care and patient safety by affecting the ability of healthcare professionals to fulfil their responsibilities in critical patient care (Emergency Care Research Institute n.d.; Ruppel et al. 2018; Sendelbach and Funk 2013).

In addition, when the literature is examined, it is possible to come across study results that suggest that anxiety triggers misophonia in a person or affects the severity of misophonia (Siepsiak et al. 2020) and that there is no relationship between anxiety and misophonia severity (Gül Kenar et al. 2022). Our literature review revealed no studies with participants consisting entirely of intensive care nurses. While the literature acknowledges that not every sound triggers misophonia, there is also uncertainty about which sounds trigger misophonic behaviours. This lack of clarity in the literature raises the question of how different sound sources in intensive care units affect staff. Obtaining data on how different sounds in intensive care affect nurses working in these units is crucial for clarifying the existence of a problem that could compromise patient safety and for developing solutions. In this context, these conflicting study results suggest that it is necessary to investigate whether there is a relationship between the severity of misophonic emotions in intensive care nurses, their personality traits, and anxiety‐provoking situations in their personal lives. The aim of this study is to investigate whether there is a relationship between the personality traits of nurses working in intensive care units, their reactions to sounds and the care provided to the patient. In addition, it was aimed to contribute to the data gap in the literature with this study data.

## 2 Hypotheses

*There is no relationship between responses of nurses working in intensive care units, which to sounds and the care behaviours provided to the patient*.

*There is relationship between responses of nurses working in intensive care units, which to sounds and the care behaviours provided to the patient*.

## 3 Materials and Methods

The research is descriptive, cross‐sectional, and relational study type. The universe of the research consisted of all the nurses working in the paediatric and adult ICU in a Sivas Cumhuriyet University Application and Research Hospital. The sample size of the study was determined by the sample calculation formula of the known universe (*S* = Nt2 p.q/*d*2(*N*‐1) + *t*2p.q) (Kılıç 2012). The sample of the study consisted of 131 nurses who agreed to participate in the study without any limitations. The sample was selected by the stratified random sampling method. The fact that intensive care nurses have roles in both patient treatment and patient care causes them to spend more time with patients and therefore to be exposed to more noise. Therefore, the sample of the study consists of intensive care nurses. In the Sivas Cumhuriyet University Application and Research Hospital there have a total of 10 different ICU from 3 different types of intensive care, including paediatric, newborn, and adult (Anaesthesia ICU, Coronary ICU, Cardiovascular Surgery ICU, General Surgery ICU, Neurology ICU, Neurosurgery ICU, Thoracic Surgery ICU, Internal Medicine ICU, Paediatrics ICU, and Neonatal ICU). Study data were obtained by completing the participant information form, misophonia scale, and caring behaviours‐24 scale from nurses working in these units.

Intensive care units in the institution where the study was conducted differ in that they have different bed capacities and varying numbers of employees, serving different patient and disease groups. However, all ICU are units with the technical equipment and competence to provide care and treatment to tertiary intensive care patients. Nurses working in the ICU work in situations that may cause stress, such as the number of patients, clinical conditions of the patients, etc., which may vary between units. All ICUs were included in the study because they were similar among nursing staff. This study followed the Strengthening the reporting of observational studies in epidemiology (STROBE) reporting guidelines.

### 3.1 Research Inclusion Criteria

Intensive care nurses who could speak and understand Turkish and had a written consent form participated in the study voluntarily.

### 3.2 Research Exclusion Criteria

It was planned to exclude unfilled or incompletely filled questionnaires from the study. However, since there were no missing or unanswered forms, all questionnaires were included in the study.

### 3.3 Application of Data Collection Tools

After obtaining permission from the institution for the study, the nurses who accepted to participate in the study filled out the participant information form about their socio‐demographic characteristics, the Misophonia scale and the care behaviours‐24 scale, and the data were obtained. It was explained to the participants that the concept of misophonia, which is not very well known and may impair the quality of care of the study, will contribute to increasing the quality of nursing care. The researchers met with the intensive care nurses in the study setting, provided information about the study, and obtained written consent from those who volunteered to participate. The forms were handed over to the intensive care nurses in envelopes for them to complete in hard copy. The intensive care nurses completed the forms independently within two days and returned them to the researchers in sealed envelopes at the end of the two‐day completion period. The study was conducted between September 1, 2023 and September 15, 2023.

### 3.4 Introductory Information Form

It is the form that contains the information about the participant, which includes questions such as the nurse's age, gender, education level, marital status, number of children, type of work, weekly working time. In our study, we tried to obtain data on some demographic characteristics of the participants that may cause anxiety in their lives to obtain data on whether there is a relationship between anxiety and misophonic reactions. For this purpose, it was questioned whether the participants were married, whether they had children, their socio‐economic status, their working hours and positions at work, whether they chose the nursing profession voluntarily, their satisfaction status, etc. In addition, the personality traits of the participants regarding their coping situations and their educational status were also questioned. In addition, in our study, participants were questioned about their personality traits and educational status in order to obtain data on their coping with stress. It is thought that coping is related to personality structure.

### 3.5 Misophonia Scale

The Misophonia Scale, developed by Wu et al. (2014), measures the symptoms of misophonia and the emotions and behaviours developed in exposure to triggering sounds. The original scale consists of two factors. Misophonia symptoms, which is the first factor in which the presence of misophonia symptoms is questioned, consist of seven statements. The items have a 5‐point Likert‐type response category from ‘not at all true’ (0) to ‘always true’ (4). The second factor, which measures the emotions and behaviours resulting from exposure to the triggering sound called misophonia emotions and behaviours, consists of 10 questions. These items have a 5‐point Likert‐type response category ranging from ‘never’ (0) to ‘always’ (4). The scored part of the scale is made by considering the answers given to a total of 17 statements in these two factors. There is no reverse item in the scale and the total score ranges from 0 to 68. As the scale score increases, it should be considered that the frequency of misophonia symptoms and the negative emotions and behaviours that the person develops against it increase. The statements in the last part of the scale give information about the severity of misophonia and are not included in the factor structure and scoring. The person is asked to choose the number and degree of sensitive sounds and how much they interfere with their daily life from a scale ranging from ‘1 to 15’. Markings of 7 and above in this section represent clinically significant misophonia (Wu et al. 2014). The Turkish validity of the scale was made by Deniz Sakarya and Çakmak in 2022 and the Cronbach Alpha internal consistency coefficient of the scale was found to be 0.89 (Deniz Sakarya and Çakmak 2022). In this study, it was stated that the Cronbach alpha value calculated for the total scale and sub‐dimensions was above 0.90.

### 3.6 Caring Behaviours Scale‐24

The scale was first developed by Wolf in 1981 to measure the extent and level of caring behaviours of nurses (Wolf et al. 1994). The scale was reduced to 24 items by Wu et al. (2006) and reorganised into four sub‐dimensions: assurance, respect, commitment, and knowledge‐skills. The scale was created to evaluate the nursing care process and was designed as a 6‐point Likert‐type scale. For the scale and each sub‐dimension, sub‐dimension scores between 1 and 6 are obtained by summing the item scores and dividing by the number of items. The validity and reliability study of the Turkish form of the scale was conducted by Kurşun and Kanan (2012). In the validity and reliability study, it was stated that the Cronbach's alpha value calculated for the total scale and sub‐dimensions was above 0.80 (Kurşun and Kanan 2012).

### 3.7 Evaluation of Data

Data were analysed with the IBM SPSS V25 program. Frequency and percentage were used in the analysis of sociodemographic data. The skewness and kurtosis values (+1, −1) were examined with the Kolmogorov–Smirnov test, and the conformity of the data distribution to the normal distribution was tested. Mann Whitney *U* test and Kruskall Wallis test were used to compare data that were not normally distributed. Pearson rank correlation coefficient was used to examine the relationship between variables. Since the data were not normally distributed, Pearson correlation coefficient was used to examine the relationship between variables. The significance level was taken as *p* < 0.05.

### 3.8 Ethical Aspect of Research

Before starting the study, approval was obtained from the ethics committee of Sivas Cumhuriyet University, Non‐Invasive Clinical Studies, dated 22.02.2023, and numbered 2023‐02/13. During the study process, the study was carried out by observing the rules of the Declaration of Helsinki.

## 4 Results

The study consisted of 131 intensive care nurses to participate in the study. The distribution of the participants in the study according to some introductory characteristics is given in Table 1. 69.50% of the participants are women, 83.96% are between the ages of 20–35, 71.75% are Associate/Bachelor's graduates, 55.70% have no children, 55.72% work in the Anaesthesia ICU, 65.60% weekly worked 0–40 h and 93.10% of them worked as service nurses (Table 1). It was also found that 66.40% of the participants chose the nursing profession voluntarily, 65.60% had a moderate socio‐economic level, 35.10% were aggressive, 35.18% had a perfectionist personality structure, and 68.70% sometimes felt stressed (Table 1). Table 2 shows the mean score of the Misophonia scale and the caring behaviour scale‐24. The misophonia scale misophonia symptoms total sub‐dimension mean of the participants was 14.45 ± 3.13, the misophonia feelings and behaviours total sub‐dimension mean was 14.58 ± 4.97, and the misophonia scale total mean score was 29.05 ± 7.65 (Table 2). The mean of the caring behaviours scale's assurance sub‐dimension was 2.75 ± 0.22, the mean of the knowledge and skill sub‐dimension was 2.48 ± 0.25, the mean of the sub‐dimension of respect was 2.80 ± 0.30, the mean of the loyalty sub‐dimension was 3.04 ± 0.29, and the mean total score of the caring behaviour scale‐24 was 2.77 ± 0.63 (Table 2).

**TABLE 1:** Distribution of participants by some introductory characteristics.

*n* = 131		
Characteristics	*n*	%
Gender		
Female	91	69.50
Male	40	30.50
Age (*X* = 29.86 ± 3.50)		
20–35	110	83.96
36–50	21	16.04
Education status		
Health vocational high School	25	19.10
Associate/Undergraduate	94	71.75
Degree	12	9.15
Having children		
Yes	58	44.30
No	73	55.70
Department of study		
Anaesthesia intensive care	73	55.72
Neonatal intensive care	10	7.64
Cardiovasculer intensive care	16	12.21
Surgical intensive care	22	16.79
Chest diseases intensive care	10	7.64
Satisfaction with working as a nurse		
Yes	84	64.10
No	47	35.90
Your weekly working hours		
0–40	86	65.60
41–50	45	34.40
Voluntary choice of nursing profession		
Yes	87	66.40
No	44	33.60
Your current job		
Responsible nurse	9	6.90
Service nurse	122	93.10
Socio‐economic level		
Low	45	34.40
Middle	86	65.60
Personality structure		
Shy	37	28.20
Aggressive	46	35.10
Attacker	2	1.52
Perfectionist	46	35.18
Frequency of feeling stressed		
Every time	34	26.00
Sometimes	90	68.70
Never	7	5.30

**TABLE 2:** Mean scores of misophonia scale and caring behaviours scale‐24 and correlation.

*n* = 131	$\overset{®}{X}$ ± SS	Min.	Max.
*Misophonia scale*			
Misophonia symptoms	14.45 ± 3.13	0	28
Misophonia emotions and behaviours	14.58 ± 4.97	0	25
Misophonia scale total score	29.05 ± 7.65	0	68
*Caring behaviours scale‐24*			
Assurance	2.75 ± 0.22	1	5
Knowledge skill	2.48 ± 0.25	1	5
Respectful	2.80 ± 0.30	1	5
Loyalty	3.04 ± 0.29	1	5
Caring behaviours scale‐24 total score	2.77 ± 0.63	1	5
	**Misophonia scale**		
**Caring behaviours scale‐24**	Correlation coefficient	Sig. (2‐tailed)	*N*
	−0.450	0.001*	131

* Correlation is significant at the 0.01 level (two‐tailed).

The correlation between the misophonia scale and the caring behaviour scale‐24 is presented in Table 2. A moderate negative correlation was found between the misophonia scale and the caring behaviour scale‐24 (*r* = −0.450, *p* = 0.001) (Table 2).

The misophonia scale sub‐dimension and total score average according to some demographic characteristics of the participants are given in Table 3. Accordingly, the misophonia feelings and behaviour sub‐dimension score average of the participants with a low socio‐economic level was found to be 14.79 ± 0.60 (*p* = 0.004). The misophonia scale mean score of the participants who had children was found to be 33.01 ± 2.40 (*p* = 0.005). When the personality traits and misophonia scale mean scores were examined, it was found that the participants with the shy personality trait had a mean score of 29.84 ± 2.87, those with the aggressive personality trait had a mean score of 26.80 ± 2.33, those with the attacker personality trait had a mean score of 26.13 ± 2.43, and those with the perfectionist personality trait had a mean score of 33.53 ± 2.54 (*p* > 0.005) (Table 3).

**TABLE 3:** Average of misophonia sub‐dimension and caring behaviours scale‐24 total scores sub‐dimension and total scores according to some demographic characteristics of the participants.

*n* = 131	Misophonia scale score averages		Caring behaviours scale‐24 sub‐dimension score averages					Caring behaviours scala‐24 total score averages
	Misophonia symptoms	Misophonia feelings and behaviours	Misophonia scale total score	Assurance	Information and skill	Respectful	Loyalty	
	*x̄* ± SS	*x̄* ± SS	*x̄* ± SS	*x̄* ± SS	*x̄* ± SS	*x̄* ± SS	*x̄* ± SS	*x̄* ± SS
*Gender*								
Female	13.91 ± 0.90	13.63 ± 0.95	27.55 ± 1.39	2.75 ± 0.23	2.50 ± 0.95	2.73 ± 0.88	3.02 ± 0.30	2.76 ± 0.87
Male	15.70 ± 0.86	16.75 ± 0.87	32.45 ± 2.70	2.75 ± 0.22	2.42 ± 0.87	2.97 ± 0.86	3.07 ± 0.29	2.81 ± 0.86
Test statistics	*t* = −1.325 *p* = 0.218	*t* = −1.844 *p* = 0.012	*t* = −1.774 *p* = 0.117	*t* = −0.027 *p* = 0.998	*t* = 0.355 *p* = 0.963	*t* = −0.946 *p* = 0.283	*t* = 0.194 *p* = 0.954	*t* = −0.265 *p* = 0.480
*Age*								
20–35	14.20 ± 0.71	14.39 ± 0.94	28.60 ± 1.39	2.80 ± 0.71	2.54 ± 0.94	2.88 ± 0.75	3.08 ± 0.73	2.83 ± 0.46
36–50	15.76 ± 1.42	15.62 ± 1.88	31.41 ± 3.29	2.51 ± 0.76	2.18 ± 0.88	2.41 ± 0.84	2.80 ± 0.81	2.47 ± 0.30
Test statistics	*t* = −0.914 *p* = 0.552	*t* = −0.578 *p* = 0.930	*t* = −0.805 *p* = 0.866	*t* = 0.994 *p* = 0.837	*t* = 1.209 *p* = 0.447	*t* = 1.532 *p* = 0.725	*t* = 0.927 *p* = 0.318	*t* = 1.351 *p* = 0.390
*Educational status*								
Vocational School of Health	15.24 ± 1.23	7.72 ± 1.55	12.41 ± 0.12	2.77 ± 0.14	2.60 ± 0.16	2.82 ± 0.15	3.15 ± 0.15	2.84 ± 0.13
Associate/Licence	14.97 ± 1.72	10.76 ± 0.88	16.95 ± 0.68	2.76 ± 0.16	2.36 ± 0.17	2.82 ± 0.17	2.90 ± 0.21	2.72 ± 0.17
Graduate	11.37 ± 0.49	9.32 ± 0.92	16.23 ± 0.48	2.67 ± 0.18	2.33 ± 0.25	2.72 ± 0.19	2.91 ± 0.26	2.66 ± 0.23
Test statistics	*F* = 2.842 *p* = 0.062	*F* = 1.060 *p* = 0.350	*F* = 2.104 *p* = 0.126	*F* = 0.064 *p* = 0.938	*F* = 0.642 *p* = 0.528	*F* = 0.065 *p* = 0.937	*F* = 0.607 *p* = 0.547	*F* = 0.266 *p* = 0.767
*Socio‐economic level*								
Low	14.79 ± 0.71	14.79 ± 0.60	29.58 ± 1.40	2.75 ± 0.13	2.53 ± 0.14	2.80 ± 0.13	3.11 ± 0.29	2.80 ± 0.12
Middle	13.82 ± 0.85	14.20 ± 0.64	28.02 ± 2.60	2.74 ± 0.17	2.37 ± 0.16	2.81 ± 0.19	2.90 ± 0.30	2.72 ± 0.16
Test statistics	*t* = 0.737 *p* = 0.182	*t* = 0.358 ***p* = 0.004** *	*t* = 0.580 *p* = 0.011	*t* = 0.053 *p* = 0.277	*t* = 0.699 *p* = 0.084	*t* = 0.015 *p* = 0.891	*t* = 0.890 *p* = 0.995	*t* = 0.414 *p* = 0.507
*Personality structure*								
Shy	14.89 ± 3.02	15.80 ± 1.82	29.84 ± 2.87	2.90 ± 0.27	2.66 ± 025	3.18 ± 0.29	3.24 ± 0.26	2.99 ± 0.24
Aggressive	13.86 ± 3.49	13.45 ± 1.45	26.80 ± 2.33	2.52 ± 0.19	2.23 ± 0.18	2.58 ± 0.20	2.87 ± 0.20	2.55 ± 0.16
Attacker	14.68 ± 2.15	12.66 ± 1.36	26.13 ± 2.43	2.98 ± 0.21	2.76 ± 0.23	2.96 ± 0.19	3.13 ± 0.22	2.96 ± 0.14
Perfectionist	16.88 ± 4.18	16.63 ± 1.59	33.53 ± 2.54	2.70 ± 0.18	2.40 ± 0.21	2.65 ± 019	3.00 ± 0.24	2.70 ± 0.20
Test statistics	*F* = 1.938 *p* = 0.127	*F* = 1.456 *p* = 0.230	*F* = 1.863 *p* = 0.139	*F* = 0.952 *p* = 0.418	*F* = 1.286 *p* = 0.282	*F* = 1.429 *p* = 0.232	*F* = 0.502 *p* = 0.682	*F* = 1.190 *p* = 0.316
*Having a child*								
Yes	15.55 ± 2.05	14.45 ± 1.40	33.01 ± 2.40	2.88 ± 0.15	2.44 ± 0.15	2.82 ± 0.13	3.02 ± 0.32	2.89 ± 0.13
No	13.58 ± 1.13	14.69 ± 1.44	25.28 ± 2.15	2.65 ± 0.14	2.51 ± 0.15	2.79 ± 0.12	3.05 ± 0.28	2.74 ± 0.14
Test statistics	*t* = 1.574 *p* = 0.104	*t* = 0.156 *p* = 0.187	***t* = 2.667** ***p* = 0.005** *	*t* = 1.058 *p* = 0.634	*t* = 0.289 *p* = 0.191	*t* = 0.097 *p* = 0.765	*t* = 0.107 *p* = 0.823	*t* = 0.318 *p* = 0.761
*Department of study*								
Anaesthesia intensive care	14.27 ± 1.19	13.95 ± 1.47	28.24 ± 2.43	2.67 ± 0.20	2.47 ± 0.19	2.63 ± 0.18	2.88 ± 0.20	2.66 ± 0.17
Neonatal intensive care	13.16 ± 2.95	14.50 ± 1.66	27.66 ± 3.69	2.92 ± 0.40	2.53 ± 0.48	3.72 ± 0.48	3.93 ± 0.29	3.21 ± 0.40
Cardiovascular intensive care	13.42 ± 1.23	12.73 ± 1.73	26.15 ± 2.64	3.02 ± 0.23	2.50 ± 0.26	2.87 ± 0.25	3.05 ± 0.25	2.88 ± 0.27
Surgical intensive care	13.69 ± 1.64	15.04 ± 2.15	28.73 ± 3.54	2.83 ± 0.27	2.46 ± 0.28	2.81 ± 0.29	3.15 ± 0.28	2.82 ± 0.21
Chest diseases intensive care	16.11 ± 1.12	16.36 ± 1.66	32.47 ± 2.29	2.57 ± 0.18	2.48 ± 0.20	2.80 ± 0.22	2.97 ± 0.22	2.72 ± 0.22
Test statistics	*F* = 0.739 *p* = 0.569	*F* = 0.688 *p* = 0.601	*F* = 0.785 *p* = 0.537	*F* = 0.592 *p* = 0.669	*F* = 0.924 *p* = 0.452	*F* = 0.952 *p* = 0.418	*F* = 0.916 *p* = 0.452	*F* = 0.432 *p* = 0.781
*Weekly working hours*								
0–40	14.55 ± 0.76	15.09 ± 1.02	15.16 ± 1.65	2.98 ± 0.12	2.51 ± 0.13	2.79 ± 0.13	3.09 ± 0.13	2.98 ± 0.11
41–50	14.26 ± 1.08	13.62 ± 1.17	13.73 ± 2.04	2.69 ± 0.19	2.42 ± 0.18	2.84 ± 0.20	2.93 ± 0.21	2.58 ± 0.18
Test statistics	*t* = 0.221 *p* = 0.831	*t* = 0.892 *p* = 0.203	*t* = 0.655 *p* = 0.465	***t* = 0.923** ***p* = 0.005**	*t* = 0.406 *p* = 0.839	*t* = 0.203 *p* = 0.136	*t* = 0.655 *p* = 0.117	***t* = 0.573** ***p* = 0.005**
*Current position*								
Service nurse	14.50 ± 0.77	14.90 ± 0.95	29.40 ± 2.73	2.75 ± 0.11	2.52 ± 0.11	2.85 ± 0.35	3.03 ± 0.22	2.79 ± 0.10
Responsible nurse	13.88 ± 0.67	10.24 ± 0.78	24.18 ± 1.66	2.76 ± 0.40	1.95 ± 0.38	2.12 ± 0.11	3.08 ± 0.11	2.52 ± 0.33
Test statistics	*t* = 0.247 *p* = 0.731	*t* = 1.512 *p* = 0.406	*t* = 1.032 *p* = 0.719	*t* = 0.019 *p* = 0.998	*t* = 1.312 *p* = 0.301	*t* = 1.634 *p* = 0.288	*t* = 0.116 *p* = 0.391	*t* = 0.719 *p* = 0.260
*Voluntary choice of nursing profession*								
Yes	15.44 ± 0.81	16.50 ± 0.95	31.94 ± 2.51	2.77 ± 0.21	2.30 ± 0.18	2.76 ± 0.24	2.91 ± 0.22	2.69 ± 0.18
No	13.97 ± 0.67	13.49 ± 0.83	27.47 ± 1.52	2.72 ± 0.12	2.60 ± 0.13	2.86 ± 0.13	3.15 ± 0.13	2.85 ± 0.11
Test statistics	*t* = 1.009 *p* = 0.677	*t* = 1.699 *p* = 0.638	*t* = 1.529 *p* = 0.996	*t* = 0.215 *p* = 0.983	*t* = 1.217 *p* = 0.064	*t* = 0.389 *p* = 0.333	*t* = 0.902 *p* = 0.875	*t* = 0.717 *p* = 0.422
*Satisfaction with working as a nurse*								
Yes	15.09 ± 0.66	17.12 ± 0.71	32.22 ± 2.66	2.91 ± 0.23	2.81 ± 0.21	3.04 ± 0.24	3.16 ± 0.75	2.99 ± 0.20
No	14.52 ± 0.70	14.65 ± 0.82	29.18 ± 2.42	2.44 ± 0.19	2.20 ± 0.18	2.60 ± 0.21	2.93 ± 0.70	2.55 ± 0.17
Test statistics	*t* = 0.313 *p* = 0.918	*t* = 1.153 *p* = 0.611	*F* = 0.849 *p* = 0.569	*t* = 1.527 *p* = 0.453	*F* = 2.156 *p* = 0.933	*F* = 1.355 *p* = 0.760	*F* = 0.738 *p* = 0.732	*F* = 1.666 *p* = 0.619
*Frequency of feeling stressed*								
Always	15.44 ± 1.22	16.50 ± 1.59	31.94 ± 2.51	2.72 ± 0.21	2.30 ± 0.18	2.76 ± 0.24	2.91 ± 0.22	2.69 ± 0.18
Sometimes	13.97 ± 0.76	13.49 ± 0.90	27.47 ± 1.52	2.77 ± 0.12	2.60 ± 0.13	2.86 ± 0.13	3.16 ± 0.13	2.85 ± 0.11
Never	15.85 ± 0.92	19.42 ± 1.12	35.28 ± 2.89	2.57 ± 0.50	1.77 ± 0.33	2.76 ± 0.10	2.22 ± 0.52	2.25 ± 0.43
Test statistics	*F* = 0.659 *p* = 0.519	*F* = 2.521 *p* = 0.084	*F* = 1.838 *p* = 0.183	*F* = 0.104 *p* = 0.901	*F* = 1.965 *p* = 0.144	*F* = 0.728 *p* = 0.485	*F* = 1.902 *p* = 0.153	*F* = 1.086 *p* = 0.341

*Note:* Values which are statistically meaningful are written in “bold”.

* *p* < 0.005.

The sub‐dimension of the caring behaviour scale‐24 and the total score average according to some demographic characteristics of the participants are presented in Table 3. The total mean score of the caring behaviour scale‐24 of the participants who worked 0–40 h a week was calculated as 2.98 ± 0.11 (*p* = 0.005) (Table 3).

## 5 Discussion

According to the data of our study, in which we investigated whether there is a relationship between the reactions of nurses working in ICUs where different sound levels and the care provided to the patient, it is possible to say that there is a moderate negative relationship between the Misophonia Scale and the Caring Behaviours Scale‐24.

When the data on the descriptive characteristics of the participants in the research are examined, it is seen that 69.50% of the participants are women and the majority (83.96%) are between the ages of 20–35. Based on these data, it is possible to say that the majority of the study participants are young. In the studies of Wu et al. (2014) and Naylor et al. (2020), the age group is young as in our study. When other studies on misophonia are examined, it is seen that there are studies conducted with different age groups. The studies of Dozier (2015) and Rosenthal et al. (2021) can be given as an example of this situation. Dozier published a study in 2015 to explain his hypothesis about the cause of emotional response to triggering stimuli. In this study by Dozier (2015), he claimed that the participants created a situation that preserves or strengthens the misophonia physical reflex with a Pavlovian conditioning mechanism and included participants of different ages in his study to prove his claim. Similarly, Rosenthal et al. (2021) included participants between the ages of 18–65 in their study to develop the Misophonia Questionnaire scale and to verify the scale psychometrically. In light of this information, it is possible to say that misophonia is a condition that can be seen in every age group. In line with the data obtained from the participants, it was determined that the mean scores of the misophonia emotions and behaviours sub‐dimension of participants with low socioeconomic status and those who had children were higher and this difference was statistically significant (*p* = 0.004), (*p* = 0.005). These factors are situations that can cause people to experience anxiety and stress, and it can be said that the presence of anxiety and stress triggers misophonic behaviours, negatively affects coping situations, and increases intolerance to stimuli, causing people to avoid stimuli. As a result of her study, Gül Kenar et al. (2022) concluded that symptoms in individuals with misophonia are triggered by certain sounds and that individuals may have to use various coping mechanisms, such as moving away from the patient and/or the scene to get rid of the disturbing stimulus, suppressing the stimulus if they cannot remove the triggering sound or move away from the environment, and responding incompatible with the current situation. In addition, the same study states that these behavioural patterns, when combined with the patient's anxiety, can impair the daily quality of life of individuals. In addition to studies in the literature that suggest that anxiety triggers misophonia in a person or affects its severity (Siepsiak et al. 2020), there are also studies that could not detect a similar relationship between anxiety and misophonia severity (Gül Kenar et al. 2022). In addition, in our study, the personality traits and educational status of the participants were questioned in order to obtain data on their coping with stress. Coping is thought to be related to personality structure. In our study, 46 participants defined themselves as having impulsive and perfectionist personality structures, and 58 of the participants scored 7 and above in the section of the misophonia scale that determines clinical severity. These findings indicate that the majority of the participants had misophonic sensitivity. Jager et al. (2020) found that individuals with misophonia were individuals with clinical perfectionism. Jakubovski et al. (2022) defined perfectionism as a personality trait associated with misophonia and suggested that the underlying reason for discomfort or intolerance to noise may be that individuals create their own realities based on perfectionist personality traits. The results of this study appear to be consistent with the results of our study.

Misophonia has selective sound sensitivity, meaning it is not triggered by all sounds. In our study, participants identified the most triggering sounds as eating and drinking‐related sounds such as slurping, smacking, and swallowing that people make with their mouths. Gül Kenar et al. (2022) identified lip smacking and throat sounds as the most common triggering sounds. During our literature review, no studies were found on misophonic triggers in which all participants were nurses. In the study of Gül Kenar et al. (2022), it is stated that one of the participants is an intensive care nurse and describes the monitor sounds in the ICU as a misophonia trigger. Similarly, Wu et al. (2014) and Zhou et al. (2017) also mentioned the presence of voice as a trigger of misophonia symptoms in their studies. Although they do not make the same definitions as the most common triggers in our study, the presence of voices is expressed as the most trigger in these studies. Edelstein et al. (2013), Jager et al. (2020), Schröder et al. (2013), and Tunç and Başbuğ (2017) found that the biggest triggers for individuals with misophonia are eating, chewing, and smacking sounds; this result supports our study results. People's responses to trigger sounds may vary due to individual differences. Dibb et al. (2021), in their study of the Misophonia Response Scale, stated that the behavioural responses of individuals to triggers may differ emotionally or physiologically between individuals. Swedo et al. (2021) make a similar conclusion and underline that how people will react behaviourally will depend on the source of the voice and the relationships.

When the mean of the caring behaviours scale‐24 assurance sub‐dimension is considered, it is seen that although there is no statistically significant difference, the mean of the sub‐dimension of commitment is numerically higher than the others. It was thought that this situation might be related to the fact that the majority of the participants chose the profession willingly and were satisfied with the profession. A moderate negative correlation was found between the misophonia scale and the caring behaviour scale‐24 (*r* = −0.450, *p* = 0.001). Based on this data, it is possible to say that the quality of care of the nurses working in the ICU with a high score on the misophonia scale is lower than that of the other nurses. In other words, it can be said that the functionality of the nurses who show misophonia symptoms in the working environment is negatively affected. Wu et al. (2014) in their study investigating the relationship between misophonia symptom frequency and functionality reached a similar conclusion to our study and reported that the functionality of 52.1% of the participants who showed clinically significant misophonia symptoms was negatively significantly affected in the work and school environment.

## 6 Conclusion and Recommendations

In light of the data we obtained from the study, it is possible to say that nurses with misophonic behaviours may be negatively affected by the sounds in the intensive care environment and their productivity may decrease, and this may negatively affect the quality of patient care. For this reason, it is recommended that different studies be conducted to support the results of the study and that data on the relationship between misophonic behaviours and the quality of patient care be increased. Thus, nurses working in ICU should be identified with misophonic findings and provided with training on methods of coping with misophonia, etc., and their coping levels should be increased. In this way, both the work efficiency of nurses will be increased and care deficiencies will be prevented, thus increasing the quality of patient care and patient safety. Misophonia is known that there is a generalisation and comparison problem between research results due to differences in evaluating misophonia. Therefore, it is recommended to increase the number of studies on misophonia. This will increase data saturation and contribute to the generalisability of the results.

## 7 Limitations of the Research

There are some limitations of the research. The fact that misophonia is a condition affected by many individual and environmental factors makes the design of study methods difficult. Our study was conducted with participants from different ICUs in a hospital, and the majority of the participants were nurses in a single ICU. Therefore, its generalisability is limited. Another limitation of the study is that differences in the evaluation of Misophonia create problems in generalisation and comparison among the research results.

## 8 Relevance for Clinical Practice

This study examined the effects of intensive care nurses on sounds in intensive care and the relationship between the effects of sounds and patient care. It can be said that the functionality of nurses showing misophonia symptoms in the work environment is negatively affected. In order to increase the functionality of nurses in the work environment, to increase the quality of care and therefore to increase patient safety, the sound sources in the environment should be managed correctly and the sound level should be reduced.

According to the research results, it was determined that participants with perfectionist personality traits were more affected by sounds. Considering that coping is related to personality structure, it is recommended that training and seminars be organised to strengthen nurses' coping methods. In this way, nurses' tolerance to sound is increased, their insensitivity to their work is prevented, and the quality of patient care and patient safety is increased.

It is known that there is a generalisation and comparison problem among research results due to differences in evaluations on misophonia. Therefore, it is recommended to increase the number of studies on misophonia. This will increase data saturation and contribute to the generalisability of the results.

## Author Contributions

**Zuhal Gülsoy:** concept, design, consultancy, data collection and/or data processing, analysis and/or interpretation, source scanning, writing of the article, critical review. **Tuba Karabey:** concept, consultancy, analysis and/or interpretation, source scanning, writing of the article, critical review.

## Funding

The authors have nothing to report.

## Ethics Statement

Before starting the study, approval was obtained from the ethics committee Sivas Cumhuriyet University from the Non‐Interventional Clinical Research Ethics Committee dated 22.02.2023, and numbered 2023‐02/13. During the study process, the study was carried out by observing the rules of the Declaration of Helsinki.

## Conflicts of Interest

The authors declare no conflicts of interest.

## Data Availability

The data that support the findings of this study are available on request from the corresponding author. The data are not publicly available due to privacy or ethical restrictions.
